# Self-reported caffeine consumption miss-matched consumption measured by plasma levels of caffeine and its metabolites: results from two population-based studies

**DOI:** 10.1007/s00394-024-03351-9

**Published:** 2024-05-04

**Authors:** Nermine Laaboub, Setareh Ranjbar, Marie-Pierre F. Strippoli, Pedro Marques-Vidal, Sandrine Estoppey-Younes, Belen Ponte, Menno Pruijm, Bruno Vogt, Nicolas Ansermot, Séverine Crettol, Frederik Vandenberghe, Peter Vollenweider, Martin Preisig, Murielle Bochud, Chin B. EAP

**Affiliations:** 1 Unit of Pharmacogenetics and Clinical Psychopharmacology, Department of Psychiatry, Centre for Psychiatric Neuroscience, Lausanne University Hospital, University of Lausanne, Prilly, Switzerland; 2 Psychiatric Epidemiology and Psychopathology Research Center, Department of Psychiatry, Lausanne University Hospital, University of Lausanne, Prilly, Switzerland; 3 Department of Internal Medicine, Lausanne University Hospital and University of Lausanne, Lausanne, Switzerland; 4 Centre for Primary Care and Public Health (Unisanté), University of Lausanne, Lausanne, Switzerland; 5 Division of Nephrology, Geneva University Hospitals, Geneva, Switzerland; 6 Service of Nephrology and Hypertension, University Hospital of Lausanne and University of Lausanne, Lausanne, Switzerland; 7 Department of Nephrology and Hypertension, Inselspital, Bern University Hospital, University of Bern, Bern, Switzerland; 8 School of Pharmaceutical Sciences, University of Geneva, University of Lausanne, Geneva, Switzerland; 9 Center for Research and Innovation in Clinical Pharmaceutical Sciences, University of Lausanne, Hôpital de Cery, Prilly, Lausanne, 1008 Switzerland; 10 Institute of Pharmaceutical Sciences of Western Switzerland, University of Geneva, University of Lausanne, Geneva, Switzerland

**Keywords:** Caffeine, Paraxanthine, Theophylline, Plasma levels, Self-reporting, Questionnaire

## Abstract

**Importance and objective:**

Self-reported caffeine consumption has been widely used in research while it may be subject to bias. We sought to investigate the associations between self-reported caffeine consumption and plasma levels of caffeine and its two main metabolites (paraxanthine and theophylline) in the community.

**Methods:**

Data from two population-based studies (SKIPOGH1 and 2 (*N* = 1246) and CoLaus|PsyCoLaus (*N* = 4461)) conducted in Switzerland were used. Self-reported caffeine consumption was assessed using questionnaires. Plasma levels of caffeine and its metabolites were quantified by ultra-high performance liquid chromatography coupled to a tandem quadrupole mass spectrometer.

**Results:**

In both studies, mean log plasma levels of caffeine and its two metabolites were over 6.48 (plasma levels = 652 ng/ml) when no caffeine consumption was reported. Subsequently, nonlinear associations between log plasma levels and self-reported caffeine consumption were observed in SKIPOGH, with a change of the slope at 3–5 cups of espresso per day in SKIPOGH1 but not SKIPOGH2. In CoLaus|PsyCoLaus, increased daily consumption of caffeinated beverages was associated with increased log plasma levels with a change of the slope at 3 cups. In both studies, declared caffeine consumption higher than 3–5 cups per day was not associated with higher plasma levels of caffeine and its metabolites.

**Conclusion:**

Self-reports of no or low caffeine consumption and consumption of more than 3–5 cups of coffee should be interpreted with caution, with possible under- or over-estimation. Quantifying plasma levels of caffeine and its metabolites may contribute to a better estimation of caffeine intake.

**Supplementary Information:**

The online version contains supplementary material available at 10.1007/s00394-024-03351-9.

## Introduction

Self-reporting is a widely used approach in medical and social research to quantify exposure, resource use, or satisfaction [1]. Self-reporting is, in general, performed by asking participants to fill in a questionnaire or survey, or to respond directly to questions from an investigator, transcribing the answers without interfering. However, because of its subjective nature, unlike laboratory tests, it is often argued that self-reporting is unreliable and subject to various biases [1, 2]. Self-reported measures have been widely used in health research, including exposure to caffeine, the most widely used legal psychostimulant in the world [3]. Indeed, caffeine exposure has been the subject of intensive work regarding its health effects. For instance, high caffeine consumption has been associated with dyslipidemia and can lead to sleep disorders [4–8] or caffeine intoxication called caffeinism [9, 10]. On the other hand, caffeine is also known to have beneficial effects, including weight loss, increased athletic performance, and reduced risks of Alzheimer and Parkinson diseases, as well as all-cause mortality [11–15]. Studies have identified some demographic and clinical factors associated with increased caffeine consumption, namely female sex, advanced age, smoking, and increased CYP1A2 activity, the enzyme responsible for more than 95% of primary caffeine metabolism [16–19]. Noteworthy, the vast majority of studies use self-reported caffeine consumption as exposure measurement, which may be subject to bias due to random or systematic reporting errors. Indeed, under- or overestimation of exposure may occur due to the variability of caffeine content in beverages/foods, but also ignorance of its existence in different foods/drinks or drugs, forgetfulness or recall bias, as well as intentional misreporting. On the other hand, the assessment of caffeine and its metabolites in plasma could allow a more objective and accurate measurement of exposure.

The main objective of the present study was to investigate the association between self-reported caffeine consumption and plasma levels of caffeine and its two main metabolites (paraxanthine and theophylline) in two population-based studies conducted in Switzerland.

## Methods

### Study design, population and setting

#### SKIPOGH

The Swiss Kidney Project on Genes in Hypertension “SKIPOGH” study is a family- and population-based study exploring genetic and environmental determinants of blood pressure. Detailed methods have been previously published [20]. Briefly, participants aged 18 to 90 years were recruited in three cantons of Switzerland (Bern (*N* = 290), Geneva (*N* = 425), and Vaud (*N* = 414)) from November 2009 to 2013 (SKIPOGH1) and from 2012 to December 2016 (SKIPOGH2), with 87% of individuals participating in both study waves. The ethics committees of Lausanne University Hospital, Geneva University Hospital, and the University Hospital of Bern approved the SKIPOGH study with participants giving a written informed consent.

#### CoLaus|PsyCoLaus

The data of the present paper stem from the first follow-up of CoLaus|PsyCoLaus a population-based, single-center cohort study designed to investigate mental disorders and cardiovascular risk factors in the community. Detailed methods have been previously published [21, 22]. Briefly, a total of 6734 individuals aged 35 to 75 years were randomly selected between 2003 and 2006 according to the civil register from the residents of the city of Lausanne, Switzerland. After the baseline assessment, the first follow-up evaluation took place between 2009 and 2013. All participants gave written informed consent, and the local Institutional Ethics Committee approved the study.

### Caffeine exposure measurements

#### Self-reported caffeine consumption

##### SKIPOGH

Participants were asked to fill in a questionnaire on the frequency of consumption of caffeinated beverages (Supplementary Table 1). First, the reported number of espresso drinks consumed per day was quantified by considering question 2 in Supplementary Table 1. Second, as caffeine is present in different beverages in different amounts, in order to consistently quantify caffeine exposure, a conversion was made to the estimated number of equivalent 60 ml espresso cups consumed per day (see supplementary Table 2 for more details). Participants who reported or estimated consuming more than 10 (9 and 5 participants in SKIPOGH1 and SKIPOGH 2, respectively) and 25 cups (21 and 16 participants in SKIPOGH 1 and SKIPOGH 2, respectively) of espresso per day, respectively, were excluded from analyses to avoid the leverage effect.

##### CoLaus|PsyCoLaus

First, frequency of coffee consumption (excluding decaffeinated coffee) was reported through the food frequency questionnaire with seven possible answers completed at the first follow-up of the study: “None in the last 4 weeks”; “once a month”; “2–3 times a month”; “1–2 times a week”; “3–4 times a week”; “one cup a day”; “two cups or more a day” [23]. Given the small proportion of participants reporting a frequency of consumption of “once per month” and “2–3 times a month”, these were consolidated into “1–3 times per month”. Second, the frequency of caffeinated beverage consumption was assessed through the question “How many cups of caffeinated beverages do you consume each day?”, with four possible answers: “None”, “1–3 per day”, “4–6 per day”, “more than 6 per day”.

#### Plasma caffeine and its metabolites levels (SKIPOGH and CoLaus|PsyCoLaus)

After an overnight fasting, blood sampling was performed in the first follow-up of CoLaus|PsyCoLaus study and both waves of SKIPOGH study. Plasma samples for analysis of caffeine, paraxanthine and theophylline were stored at −20 °C, and were quantified by ultra-high performance liquid chromatography (Waters ACQUITY UPLC system) coupled to a tandem quadrupole mass spectrometer (Waters TQD with electrospray ionization or Waters Xevo TQ-S with UniSpray ion source). The method was validated according to international guidelines using a stable isotope-labeled internal standard for each analyte. Limit of quantification for all analytes was 5 ng/ml. The full method description is available on request.

### Covariates

Covariates associated with caffeine and its metabolites based on a priori knowledge were identified, namely: age, sex, smoking (currently), body mass index (BMI), kidney function (evaluated using the glomerular filtration rate calculated using the Chronic Kidney Disease - Epidemiology Collaboration formula [24]) and time spent between blood drawing and last caffeine intakes (only in SKIPOGH).

### Statistical analyses

Clinical and laboratory characteristics of SKIPOGH (1 and 2) and CoLaus|PsyCoLaus participants were reported as numbers and percentages, or median and interquartile range (IQR), as appropriate. Plasma levels of caffeine, paraxanthine and theophylline were summed to more accurately estimate the exposure to caffeine and its metabolites. This measure allows to reduce heterogeneity due to different time intervals between caffeine intake and blood sampling. Then, the sum of these plasma levels was log-transformed to better comply with the assumption of normality necessary for the estimation of our linear regression. Basic correlations analyses were conducted using Pearson correlation (SKIPOGH) and Dunn’s tests (CoLaus|PsyCoLaus) to examine the relationship between self-reported caffeine consumption and log plasma levels. Linear regression models were fitted to investigate the association between self-reported daily caffeine consumption and plasma levels of caffeine and its two metabolites in the first and second waves of SKIPOGH and in CoLaus|PsyCoLaus, respectively. Linear regression models were controlled for clinical covariates selected by the backward procedure based on significant p-value (*p* < 0.05) at each step, and were conducted twice in each study, considering the reported number of espresso and the estimated number of equivalent 60 ml espresso in SKIPOGH and coffee cups and caffeinated beverages, in CoLaus|PsyCoLaus. In SKIPOGH, models were fitted by ignoring (Model 1) and considering (Model 2) the structural change at some breakpoints identified graphically and/or using the chow test [25]. Subsequently, Models 1 and 2 were compared using ANOVA tests to assess the importance of considering the change of slope.

All analyses were performed using Stata 16.0 (StataCorp; College Station, Texas), and the R environment for statistical computing version 4.1.1. The significance level was considered at p-value ≤ 0.05.

## Results

### SKIPOGH

Table 1 displays the cohort characteristics. Median plasma level of caffeine and its two metabolites was 1492 ng/ml in SKIPOGH1 and decreased to 1407 ng/ml in SKIPOGH2. Median times between last self-reported caffeine consumption and blood sampling were 19 and 20 h in SKIPOGH 1 and 2. Moreover, participants reported consuming a median of two cups of espresso per day in both study waves, whereas the estimated number of equivalent 60 ml espresso cups consumed per day was 5 in the first wave and decreased to 4 in the second wave. No participant had unquantifiable plasma levels of caffeine and its metabolites (plasma levels ≤ 5 ng/ml) in both study waves. Finally, in both study waves, positive correlations were observed between plasma levels of caffeine and its metabolites and self-reported caffeine consumption, considering the reported daily espresso consumption (r_SKIPOGH1_ = 0.29, p_SKIPOGH1_ = < 10^− 4^; r_SKIPOGH2_ = 0.25, p_SKIPOGH2_ = < 10^− 4^) and the estimated 60 ml espresso consumption (r_SKIPOGH1_ = 0.12, p_SKIPOGH1_ = 0.004; r_SKIPOGH2_ = 0.16, p_SKIPOGH2_ = < 10^− 4^), respectively.

**Table 1:** Cohorts’ characteristics

	SKIPOGH 1 (*N* = 535)	SKIPOGH 2 (*N* = 711)	CoLaus| PsyCoLaus (*N* = 4461)
Age (years; median [IQR])	48 [32–61]	52 [37–64]	57 [49–66]
*Sex (N (%))*			
Male	272 (51)	358 (50)	2065 (46)
Female	263 (49)	353 (50)	2396 (54)
*Current smokers (N (%))*			
Yes	132 (25)	199 (28)	931 (21)
No	400 (75)	508 (72)	3524 (77)
Unknown (N)	3	4	6
Body mass index (kg.m^− 2^; median [IQR])	24.5 [22.1–27.3]	24.8 [22.3–28.2]	25.6 [23.0-28.5]
Unknown	1	1	36
Kidney function (ml/min/1,73 m²; median [IQR])^a^	97 [84–108]	93 [80,105]	82 [72,94]
Unknown (N)	3	2	
Caffeine (ng/ml; median [IQR])	642 [232–1384]	570 [226–1226]	751 [316–1578]
Log caffeine (ng/ml; mean (SD))	6.26 (1.38)	6.19 (1.31)	6.44 (1.35)
Paraxanthine (ng/ml; median [IQR])	706 [341–1192]	659 [317–1149]	832 [440–1349]
Log paraxanthine (ng/ml; mean (SD))	6.34 (1.05)	6.34 (0.98)	6.52 (1.07)
Theophylline (ng/ml; median [IQR])	132 [71–223]	126 [71–214]	149 [81–236]
Log theophylline (ng/ml; mean (SD))	4.73 (0.96)	4.73 (0.89)	4.83 (0.99)
Caffeine + paraxanthine + theophylline (ng/ml; median [IQR])	1492 [715–2858]	1407 [651–2642]	1799 [877–3203]
Log caffeine + paraxanthine + theophylline (ng/ml; mean (SD))	7.14 (1.15)	7.10 (1.08)	7.31 (1.15)
Time (hours; median [IQR]) ^b^	19 [14–24]	20 [14–23]	
Unknown (N)	62	47	
Espresso cups per day ^c^ (median,[IQR])	2 [1–4]	2 [1–4]	
Espresso 60 ml cups per day ^d^ (median,[IQR])	5 [2–9]	4 [2–7]	
*Coffee consumption (N (%))*			
None in the last 4 weeks			425 (10)
1–3 times a month			134 (3)
1–2 times a week			129 (3)
3–4 times a week			230 (5)
1 time a day			1032 (23)
Two or more a day			2511 (56)
*Cups of caffeinated beverages (N (%))*			
None per day			314 (7)
1–3 per day			2930 (66)
4–6 per day			1021 (23)
More than 6 per day			164 (4)

*Abbreviations* IQR = interquartile range; kg = kilograms; m = meter; min = minute; ml = milliliter; N = number; ng = nanogram; SD = standard deviation

^a^Evaluated using glomerular filtration rate calculated using Chronic Kidney Disease - Epidemiology Collaboration formula

^b^Time between last caffeine consumption and blood intake

^c^Estimated by considering the answer to question 2 of the questionnaire (supplementary Table 1)

^d^Estimated by considering all caffeinated drinks

Although 8% (*N* = 40) and 7% (*N* = 50) of participants in SKIPOGH1 and 2, respectively, reported no caffeine consumption (estimated number of 60 ml espresso cup consumed per day of 0), log plasma levels of caffeine and its two metabolites averaged greater than 6 (plasma levels greater than 403ng/ml) in both waves. Among participants reporting no caffeine consumption in the whole cohort, 2% (*N* = 11) and 2% (*N* = 13) had log plasma levels higher than the means in the first and second waves, respectively (Supplementary Fig. 1).

In addition, the multivariable analyses revealed intercepts ranging from 6.48 to 7.18, implying that when no caffeine consumption was reported, plasma levels averaged between 652ng/ml and 1313ng/ml (Table 2). When the change of slope was not considered (Table 2 - Model 1), each additional cup of 60 ml espresso reported was associated with a 3% increase in log plasma levels of caffeine and its two metabolites in both study waves. Thus, for a reported consumption of one 60 ml espresso per day, plasma levels in SKIPOGH1 and 2 were 1043 (exp(6.92 + 0.03)) and 672 (exp(6.48 + 0.03)) ng/ml, respectively. When, considering the change of slope at 5 and 6 cups in SKIPOGH1 and 2 (Table 2 - Model 2), respectively, for a consumption of 5 and 6 cups of 60 ml espresso, log plasma levels of caffeine and its two metabolites increased by 16% and 6% from no reported consumption, in the first and second waves of the study, respectively. Thus, plasma levels of caffeine and its two metabolites were 1541 (exp(7.18 + 0.16)) ng/ml for a SKIPOGH1 participant who reported consuming an equivalent of 5 cups of 60 ml espresso per day, and 728 (exp(6.53 + 0.06)) ng/ml for a SKIPOGH2 participant who reported consuming an equivalent of 6 cups of 60 ml espresso per day. A report of higher caffeine consumption was not endorsed by a significant increase in log plasma levels of caffeine and its two metabolites in both study waves (Table 2). Of note, the change of slope was statistically significant in SKIPOGH1 (p-value of ANOVA test < 10^− 3^, data not shown) while only a trend was found in SKIPOGH 2 (p-value of ANOVA test = 0.08, data not shown).

**Table 2:** Association between log plasma levels of caffeine and its metabolites and the estimated 60 ml espresso cups daily consumed in SKIPOGH (considering all caffeine source)

		Caffeine + paraxanthine + theophylline plasma levels ^a^			
		Model 1		Model 2	
	Predictors	Estimates (95% Confidence Interval)	p-value	Estimates (95% Confidence Interval)	p-value
SKIPOGH 1 (*N* = 428)	Intercept	6.92 (6.51; 7.32)	**< 10** ^**− 3**^	7.18 (6.64; 7.71)	**< 10** ^**− 3**^
	60 ml espresso ^b^	0.03 (0.01; 0.05)	**0.007**	0.00 (−0.03; 0.04)	0.89
	Change of slope			−0.62 (−1.08; −0.16)	**0.008**
	Change of slope ˣ 60 ml espresso ^b^			0.16 (0.08; 0.25)	**< 10** ^**− 3**^
SKIPOGH 2 (*N* = 610)	Intercept	6.48 (6.14; 6.82)	**< 10** ^**− 3**^	6.53 (6.06; 7.00)	**< 10** ^**− 3**^
	60 ml espresso ^b^	0.03 (0.01; 0.04)	**< 10** ^**− 3**^	0.02 (−0.01; 0.05)	0.14
	Change of slope			−0.19 (−0.58; −0.21)	0.36
	Change of slope ˣ 60 ml espresso ^b^			0.06 (0.00; 0.12)	**0.04**

Model 1 was not adjusted for the change of slope, while Model 2 was adjusted for the change of slope at 5 and 6 cups of 60 ml espresso per day in SKIPOGH1 and SKIPOGH2, respectively. All models were adjusted for age, smoking and time spent between last caffeine intake and blood intake

SKIPOGH 1 and 2 had 3% increase in log plasma levels after each 60 ml espresso cup, ignoring the change of the slope (Model 1). Plasma SKIPOGH1 and 2 levels were 1043 (exp(6.92 + 0.03)) and 672 (exp(6.48 + 0.03)) ng/ml for one 60 ml espresso per day

Considering the change of the slope (Model 2), log plasma levels increased by 16% for 5 60 ml espresso cups in SKIPOGH1 and 6% for 6 in SKIPOGH2. Thus, SKIPOGH1 plasma levels were 1541 (exp(7.18 + 0.16)) after 5 60 ml espresso cups, whereas SKIPOGH2 plasma levels were 728 (exp(6.53 + 0.06)) after 6 cups. In both waves, more espresso intake did not raise log plasma caffeine and its metabolites

^a^Log transformed

^b^The estimated number of 60 ml espresso cups consumed per day

ˣInteraction term

**Fig. 1:**
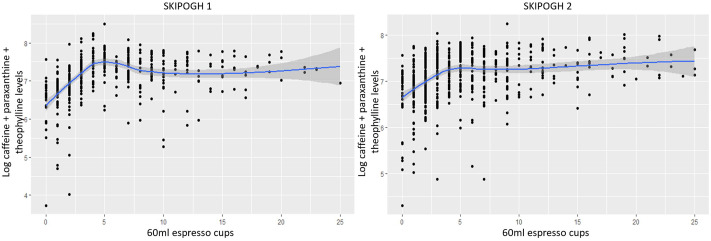
Prediction of log caffeine and its metabolites plasma levels in SKIPOGH with the change of slopes

Figure 1 shows the predicted log plasma levels of caffeine and its metabolites in function of the number of 60 ml espresso cups consumed per day. Thus, increasing the number of equivalent 60 ml espresso cups up to approximately 5 and 6 cups was associated with an increase in log plasma levels of caffeine and its metabolites in the first and second study waves, respectively. Beyond that, a small decrease followed by a plateau effect was found in SKIPOGH 1, while a small increase was found in SKIPOGH 2.

Considering only the reported espresso consumption, similar results to those obtained when considering all caffeine sources were observed, with a significant change of slope at 3 cups of espresso per day in SKIPOGH1 and a trend toward a change of slope at 4 cups in SKIPOGH2 (see supplementary Table 3 for more details).

Lastly, as shown in Supplementary Fig. 2 and Supplementary Table 4, increased log paraxanthine/caffeine ratios, which reflects CYP1A2 activity, were associated with increased reported caffeine consumption (considering only espresso consumption or all caffeine source) in both SKIPOGH waves. Thus, with increasing CYP1A2 activity, self-reported caffeine consumption increased, especially above 5 cups of 60 ml espresso per day. In addition, median paraxanthine/caffeine ratios were higher in participants consuming at least 5 cups of 60 ml espresso per day compared to those with lower espresso consumption (p-value _SKIPOGH1_ = 0.0002, p-value _SKIPOGH2_ = 0.08), while the reverse was true for median time spent from last caffeine intake to blood draw (p-value < 10^− 4^). Thus, the median time between the last reported caffeine intake and blood draw was 20 h in participants consuming less than 5 cups of espresso in both waves, whereas it was 15 h in those consuming at least 5 cups per day in both waves.

### CoLaus|PsyCoLaus

Median plasma levels of caffeine and its metabolites was 1799 ng/ml. A total of 23% and 56% reported consuming one and two or more cups of coffee per day, and 66%, 23% and 4% reported consuming 1–3, 4–6 and more than 6 cups of caffeinated beverages per day, respectively (Table 1). Of note 23 (< 1%) participants had unquantifiable plasma levels of caffeine and its metabolites (≤ 5ng/ml) of whom 4, 4, 2, and 1 reported consuming 1–3 coffee per month, 1–2 per week, 1 per day, and at least two coffee per day, respectively. Finally, of these 23 participants, 16 reported not consuming caffeinated beverages daily, while 5 and 2 reported consuming 1–3 and 4–6 caffeinated beverages per day.

**Fig. 2:**
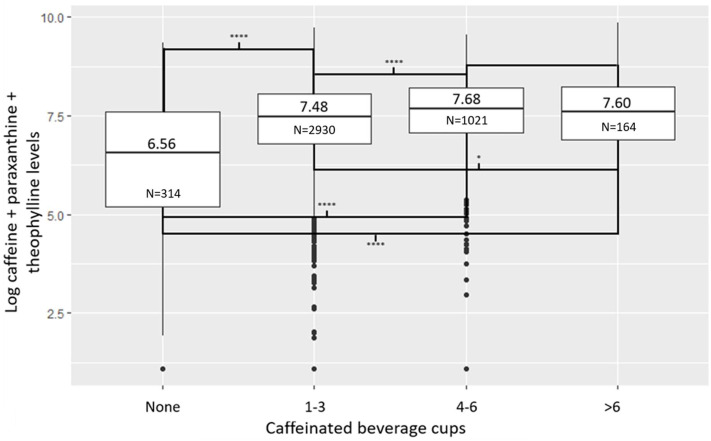
Distribution of log plasma levels of caffeine and its two metabolites according to the number of caffeinated beverage cups consumed daily in CoLaus|PsyCoLaus (*N* = 4423). Comparison were conducted using Dunn’s test. ****: p-value ≤ 10^− 4^;*: p-value ≤ 0.05

Figure 2 shows that, in participants reporting no daily consumption of caffeinated beverages, the median of log plasma levels of caffeine and its two metabolites was 6.56 (plasma levels = 706 ng/ml), whereas in those who reported consuming more than 6 cups per day, the median of log plasma levels was 7.60 (plasma levels = 1999 ng/ml). In addition, the difference in median plasma levels between participants reporting no daily consumption of caffeinated beverages and those reporting consuming 1–3, 4–6, and more than 6 cups per day was highly significant. In contrast, the difference in median plasma levels between participants reporting consuming 1–3 and more than 6 cups per day was weaker, while no significant difference was observed between participants reporting consuming 4–6 when compared to those reporting consuming more than 6 cups per day. Finally, the distribution and pairwise comparisons of median log plasma levels of caffeine and its two metabolites in relation to self-reported coffee consumption were presented in Supplementary Fig. 4 and Supplementary Table 6. The findings indicate that log plasma levels increased positively with self-reported coffee consumption, with the highest frequency reported in the questionnaire being two cups per day.

The multivariable analysis intercept showed a mean log plasma levels of caffeine and its two metabolites of 6.93 (plasma levels = 1023ng/ml; Table 3) for a consumption of more than 6 cups of caffeinated beverage per day. Therefore, compared with participants reporting consuming more than 6 cups per day, those reporting no consumption of caffeinated beverages daily and those reporting consuming 1–3 cups per day had significantly lower log plasma levels. However, and as previously shown, no significant difference was observed between those reporting consuming 4–6 cups and those reporting consuming more than 6 cups of caffeinated beverage per day, highlighting a change of slope at 3 cups per day. Similar results were found when considering only coffee consumption (see supplementary Tables 5 and 6, and supplementary Fig. 4 for more details).

**Table 3:** Association between log plasma levels of caffeine and its metabolites and the reported caffeine consumption in CoLaus|PsyCoLaus

		Caffeine + paraxanthine + theophylline plasma levels^a^	
	Predictors	Estimates (95% confidence interval)	p-value
Caffeinated beverage consumption (*N* = 4423)	Intercept (6 or more cups per day)	6.93 (6.69; 7.17)	**< 10** ^**− 3**^
	*Caffeinated beverage* ^*b*^		
	None per day	−1.43 (−1.64; −1.22)	**< 10** ^**− 3**^
	1–3 per day	−0.31 (−0.48; −0.14)	**< 10** ^**− 3**^
	4–6 per day	−0.04 (−0.22; 0.14)	0.65

Model was adjusted for age and smoking

^a^Log transformed

^b^Compared to those who reported consuming more than 6 cups per day

Finally, caffeinated beverages consumption became more frequent as paraxanthine/caffeine ratios increased (Supplementary Fig. 3 and Supplementary Table 4). However, no significant difference of CYP1A2 activity was observed between participants reporting consuming 4–6 cups of caffeinated beverages per day and those reporting consuming more than six (Supplementary Table 4).

## Discussion

Self-reporting has a high potential risk for bias by under-or overestimation of exposure, which could distort results, conclusions, and recommendations, especially in medical research. Although such biases can affect results, they are still often ignored in practice [1], raising the important issue of identifying and addressing them. In the present study, data from two population-based studies were used to investigate whether self-reported caffeine consumption, which is widely used in studies, correlated with plasma levels of caffeine and its two main metabolites. Our null hypothesis was that participants self-reporting no caffeine consumption would exhibit undetectable plasma levels of caffeine and its metabolites (i.e., plasma levels less or equal to 5ng/ml for each metabolite). Thus, an intercept indicating a mean log plasma level less than 2.71 (plasma levels less than 15ng/ml) was expected in the multivariable analyses. Subsequently, a positive association was expected between the number of self-reported cups of coffee/caffeinated beverage consumed daily and plasma levels of caffeine and its two main metabolites, which was rejected by the results showing that self-reported caffeine consumption could be a source of bias.

In both SKIPOGH and Colaus|PsyColaus, for subjects reporting no caffeine consumption (representing between 7 and 12% of both cohorts), plasma levels of caffeine and its two metabolites were over 652ng/ml. Thus, such participants likely underestimated their caffeine consumption, intentionally or not. In addition, some CoLaus|PsyCoLaus participants, representing less than 1% of the whole cohort, had undetectable plasma levels of caffeine and its two metabolites despite reporting daily caffeine consumption, resulting in an overestimation of their caffeine intake.

Furthermore, in both studies, the associations between increasing log plasma levels of caffeine and its two metabolites and the number of reported coffee/caffeinated beverages cups were not linear. Indeed, in SKIPOGH 1 and considering only the reported espresso consumption, for up to three espresso cups consumed per day, the increase in the number of cups was in agreement with the increase in plasma levels of caffeine and its two metabolites. However, above three espresso cups per day, the associations were no longer significant. Considering all caffeine sources listed in the SKIPOGH questionnaire, the change of slope was pushed from 3 to 5 cups of 60 ml espresso, however, the same results were found in the presence of a change of slope. In SKIPOGH2, the change of slope was not statistically significant, implying a positive association between plasma levels of caffeine and its two metabolites and reported espresso consumption, with a trend toward a change in the magnitude of the associations beyond a reported consumption of 4 cups of espresso. The change of slope and the change in the associations’ magnitude may be due to an overestimation of caffeine consumption as well as to the low number of individuals reporting consumption of more than 3–5 cups per day. On the other hand, although both caffeine and two main metabolites were measured, it cannot be excluded that individuals with high CYP1A2 activity metabolized and eliminated both caffeine and its metabolites more rapidly and therefore consumed more coffee. Therefore, despite high caffeine consumption (3–5 cups of espresso per day), this was no longer significantly associated with increased plasma levels of caffeine and metabolites. Of note, excessive caffeine consumption has been associated with CYP1A2 metabolic enzyme capacity saturation [26], which can lead to an accumulation of caffeine metabolites, specifically theophylline. Furthermore, several clinical and genetic factors are known for inducing or inhibiting CYP1A2 activity (e.g., oral contraceptives, smoking, some genetic variants located in the CYP1A2 gene or in other genes/regulatory regions) [27–29]. Therefore, CYP1A2 inducer users and individuals with high CYP1A2 activity should have elevated plasma levels of paraxanthine and theophylline and reduced levels of caffeine, and vice versa. However, this should have no effect on our results since we modeled the sum of the three components.

Predictions of plasma levels of caffeine and its two metabolites when increasing the number of reported daily consumption of espresso cups showed that up to about 3 cups (considering espresso consumption only) and 5 cups (considering all caffeinated beverages), positive associations were observed between the two variables. Beyond these thresholds, controversial associations were noticed, with even a slight decrease in plasma levels in SKIPOGH 1 while a slight increase was observed in SKIPOGH2. Of note, in the first wave of the SKIPOGH study, participants were not specifically informed that questions about caffeine consumption would be asked, allowing little time to adequately estimate it. As more than 87% of the participants took part in both study waves, participants in SKIPOGH2 could have better estimated their caffeine consumption the second time, remembering that such questions would be asked. Therefore, better estimation and self-reporting of caffeine consumption by SKIPOGH2 participants may explain the slight increase in plasma levels in SKIPOGH2 compared with SKIPOGH1.

The estimation of caffeine consumption by CoLaus|PsyCoLaus participants was not detailed, assessing only coffee and caffeinated beverage consumption without indicating the type of coffee/beverage or volume. Nevertheless, similar results to those of the SKIPOGH study were found. In fact, considering coffee consumption, plasma levels of caffeine and its two metabolites were high in participants reporting no coffee consumption. However, because the frequency of consumption was not detailed beyond two cups per day, a change of slope was not observed, which was the case when the consumption of all caffeinated beverages was considered. Indeed, no significant difference in plasma levels of caffeine and its two metabolites was observed between participants consuming 4 to 6 cups per day and those with more than 6 cups per day, indicating a likely change of slope at 3 cups per day.

Misreporting (voluntary or not), and/or ignorance of caffeine consumption in some foods/beverages, and/or not indicating caffeine-containing drugs in the questionnaire could explain the mismatch between plasma levels of caffeine and its metabolites and the reported caffeine consumption. Thus, the similar results of two large population-based studies with very different questionnaires on caffeine consumption highlighted the importance of objectively assessing exposure to caffeine.

The present study has several limitations. The quantification of plasma levels of caffeine and metabolites has some limitations in itself. First, the time interval between the last caffeine intake and blood sampling may have been misreported (SKIPOGH) or was unknown (CoLaus|PsyCoLaus). However, summing the plasma levels of caffeine and its two main metabolites (paraxanthine and theophylline) should reduce the influence of variable time intervals, a short interval of time leading to higher plasma levels of caffeine and lower plasma levels of paraxanthine and theophylline, the reverse being true for longer periods of time. In addition, summing the concentrations of 3 substances should allow a better estimation of the pharmacologically active molecules. It should also be mentioned that theobromine, another caffeine metabolite, was not taken into account because its main source is chocolate while the present study focused on caffeine consumption [30]. However, considering also theobromine levels did not influence our results (data not shown). Secondly, while the present study focused on two main metabolites of caffeine, 70 to 80% and 7 to 8% of caffeine being metabolized to paraxanthine and theophylline, respectively [31], other metabolites than theobromine have been described and were not quantified in the present study. Third, not all sources of caffeine or its metabolites (e.g., theophylline) were covered by the SKIPOGH questionnaire, namely some foods (e.g., cakes, pastries, breakfast cereals), over-the-counter drugs (e.g., some analgesics and bronchodilators), and some dietary supplements. Nevertheless, these foods contain negligible amounts of caffeine, and less than 3% of SKIPOGH participants reported using drugs that contained theophylline or caffeine. In addition, by examining solely self-reported espresso consumption as opposed to all sources of caffeine addressed by the questionnaire (i.e., estimated 60 ml espresso consumption), the results remained consistent. Thus, our results should not be significantly altered by incorporating additional caffeine sources. Fourth, the SKIPOGH questionnaire has not been validated, which is also the case for the vast majority of studies about caffeine consumption.

## Conclusion

Self-reported caffeine consumption may be subject to bias, with possible under- or overestimation. Reports of no consumption of caffeine and consumption of over 2 to 3 cups of coffee should be interpreted with caution. Determinations of plasma levels of caffeine and its metabolites could contribute to better estimate the relationship between caffeine consumption and the adverse or beneficial health effects of xanthines.

### Electronic supplementary material

Below is the link to the electronic supplementary material.

Supplementary Material 1

## Data Availability

CoLaus|PsyCoLaus: The data of CoLaus|PsyCoLaus study used in this article cannot be fully shared as they contain potentially sensitive personal information on participants. According to the Ethics Committee for Research of the Canton of Vaud, sharing these data would be a violation of the Swiss legislation with respect to privacy protection. However, coded individual-level data that do not allow researchers to identify participants are available upon request to researchers who meet the criteria for data sharing of the CoLaus|PsyCoLaus Datacenter (CHUV, Lausanne, Switzerland). Any researcher affiliated to a public or private research institution who complies with the CoLaus|PsyCoLaus standards can submit a research application to research.colaus@chuv.ch or research.psycolaus@chuv.ch. Proposals requiring baseline data only, will be evaluated by the baseline (local) Scientific Committee (SC) of the CoLaus and PsyCoLaus studies. Proposals requiring follow-up data will be evaluated by the follow-up (multicentric) SC of the CoLaus|PsyCoLaus cohort study. Detailed instructions for gaining access to the CoLaus|PsyCoLaus data used in this study are available at www.colaus-psycolaus.ch/professionals/how-to-collaborate/. SKIPOGH: The datasets analyzed during the current study are not publicly available due to sensitivity of the data, as it may compromise individual privacy, but may be available from Professor Murielle Bochud (main coordinator of the SKIPOGH study) on reasonable request to murielle.bochud@unisante.ch.
