# Supplementary material for: Self-reported caffeine consumption miss-matched consumption measured by plasma levels of caffeine and its metabolites: results from two population-based studies
Source: Eur J Nutr. 2024 May 4;63(5):1555–64. doi: 10.1007/s00394-024-03351-9 (PMC11329688; doi:10.1007/s00394-024-03351-9)
Supplement: Supplementary file 1 — Supplementary Material 1 [file 394_2024_3351_MOESM1_ESM.docx]

Supplementary file

Supplementary Table 1: SKIPOGH questionnaire on caffeinated beverages consumption

Consumption of caffeinated beverages

(coffee, tea, Coca Cola, cold tea, energy drinks like Red Bull, Guarana, shots)

Date of visit: SKIPOGH number : Initials : Date of birth :

1. **History of** caffeine consumption:
   1. **Have you ever consumed drinks containing caffeine? yes no**
   2. **Since what age have** you **been** drinking caffeinated beverages regularly? *Observer number of years*:
   3. **If you have had years of interruption**: for how many years in total have you been drinking caffeine regularly?
   4. **If you stopped** using caffeine, when did you stop (year)? Check here and complete the questionnaire considering the period of consumption
2. **Do you drink coffee regularly**?

| never |  |  |
| --- | --- | --- |
| 1-4 times/month |  |  |
| 1-4 times/week |  |  |
| ≥5 times/week |  |  |
| ≥1 time/day. |  | If yes, how many times/day |

- 1. **How much, on average,** do you consume each time?

| a. 1 espresso cup - 60ml | 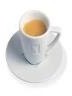 | c. 1 cup regular coffee - 140ml | 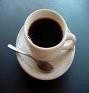 |
| --- | --- | --- | --- |
| b. 1 plastic cup - 90ml | 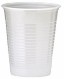 | d. 1 'mug' – 250ml | 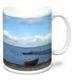 |

- 1. **What type of coffee** do you usually drink? (several answers possible)

ml

| Paper-filter dripping machine | 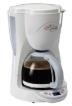 |  | Turkish/Greek coffee | 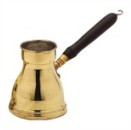 |
| --- | --- | --- | --- | --- |
| French/Italian coffee | 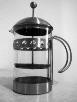 | 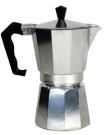 | Lyophilized coffee powder | 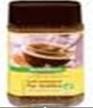 |
| Espresso-machine | 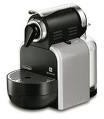 |  |  |  |

1. **Do you regularly drink caffeine-free coffee**?

| never |  |  |
| --- | --- | --- |
| 1-4 times/month |  |  |
| 1-4 times/week |  |  |
| ≥5 times/week |  |  |
| ≥1 time/day. |  | If yes, how many  times/day |

- 1. **How much, on average,** do you consume each time?

| a. 1 espresso cup - 60ml | | 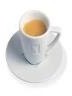 | | c. 1 cup regular coffee - 140ml | | 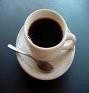 | |
| --- | --- | --- | --- | --- | --- | --- | --- |
| b. 1 plastic cup - 90ml | | 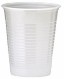 | | d. 1 'mug' (for tea for example) - 250 | | 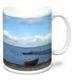 | |
|  | |  | |  | |  | |

- 1. **What type of non-caffeinated coffee** do you usually drink? (several answers possible)

ml

| Espresso-machine | 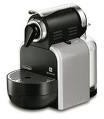 | Lyophilized coffee powder | 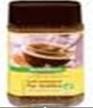 |
| --- | --- | --- | --- |
| French/Italian coffee | 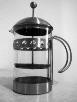 | Paper-filter dripping machine | 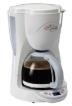 |

1. **Do you regularly drink other caffeinated beverages** (tea, Coca-Cola, cold tea, caffeinated energy drinks like Red Bull, guarana, shots)?

| never |  |  |
| --- | --- | --- |
| 1-4 times/month |  |  |
| 1-4 times/week |  |  |
| ≥5 times/week |  |  |
| ≥1 time/day. |  | If yes, how many times/day |

- 1. **What type of caffeinated beverages** and how much, **on average,** do you consume each time? (several answers possible)

| **Type of drink** | **Usual quantity** |  | **Quantity consumed (***e.g. ½ bottle or ½ can)* |
| --- | --- | --- | --- |
| Black or green tea (bag or tea ball) | e. 250ml mug | 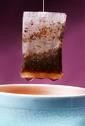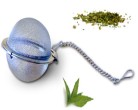 |  |
| Soft drink : Coca Cola, cold tea, etc... | f. 500ml bottle | 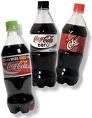 |  |
| Energy drink (Red Bull, shot, Guarana (etc..) | g. 250 ml can | 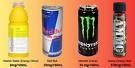 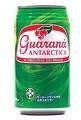 |  |

1. **Do you regularly eat dark chocolate?**

| never |  |  |
| --- | --- | --- |
| 1-4 times/month |  |  |
| 1-4 times/week |  |  |
| ≥5 times/week |  |  |
| ≥1 time/day. |  | If yes, how many times/day |

- 1. **How much, on average,** do you eat each time? squares (h. 3 squares= 5g) or in grams

Complete with the investigator, before the blood test

1. **When was the last time** you consumed caffeine (tea, coffee, coca-cola or other

caffeinated beverages such as Red Bull, dark chocolate)? date / / h

- 1. **How much** caffeinated **food did** you consume at that time? See questions 2.a, 4.a and 5.a answers a🡪:

*Observer - time of blood draw*:

**Supplementary Table 2:**  Estimation of the number of 60ml espresso daily consumed based on all caffeinated beverages

| **Caffeinated beverage** | **Amount of caffeine on milligrams (100ml)** |
| --- | --- |
| Espresso–machine ^a^ | 119 |
| Paper-filter dripping machine ^b^ | 76 |
| French/Italian coffee ^a^ | 259 |
| Turkish/Greek coffee ^c^ | 89 |
| Lyophilized coffee ^a^ | 40 |
| Black or green tea ^a^ | 13.5 |
| Soft drink (coca cola, etc.) ^a^ | 7 |
| Energy drink (Red Bull, etc.) ^a^ | 32 |

To obtain the total amount of caffeine consumed per day, the reported consumption in milliliter was converted into milligrams by calculating the milligram equivalent for each milliliter (60, 90, 140 or 250) and multiplying by the number of cups consumed per day. This total amount was then divided by 72 milligrams to obtain the equivalent number of 60ml espresso drinks consumed per day.

^a^: Rochat, C.; Eap, C.B.; Bochud, M.; Chatelan, A. Caffeine Consumption in Switzerland: Results from the First National Nutrition Survey MenuCH. Nutrients 2020, 12, 28. https://doi.org/10.3390/nu12010028

^b^: Combien de caféine y a-t-il dans une tasse de café ou de thé? | Van Houtte. Accessed September 21, 2022. https://www.vanhoutte.com/fr/faqs/produits-et-nutrition/combien-de-cafeine-y-a-t-il-dans-une-tasse-de-cafe-ou-de-the

^c^: Loison M. Quelle est la quantité de caféine dans le café et l’expresso? Umvie. Published November 22, 2019. Accessed September 21, 2022. https://umvie.com/cafeine-cafe-expresso/

**Supplementary Figure 1**: correlation between log plasma levels of caffeine and its two metabolites and the estimated number of 60ml espresso consumed daily in SKIPOGH


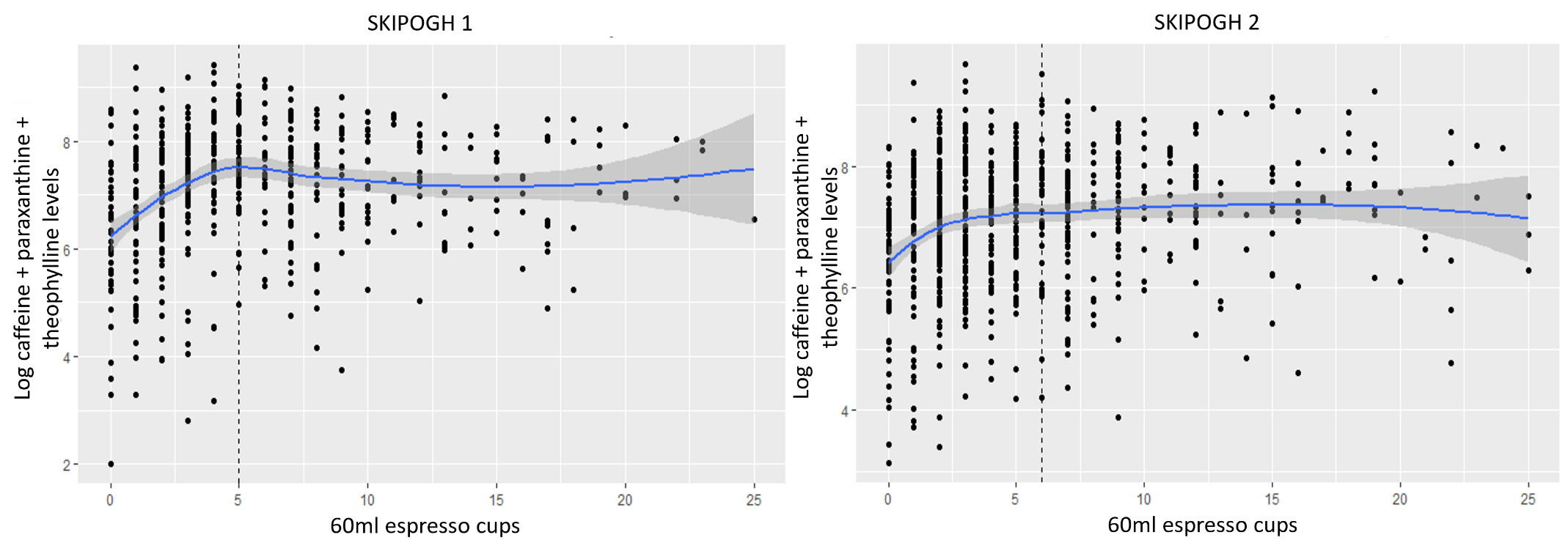


**Supplementary Table 3:** Association between log plasma levels of caffeine and its metabolites and the reported espresso daily consumption in SKIPOGH (considering only espresso consumption)

|  |  | Caffeine + paraxanthine + theophylline plasma levels ^a^ | | | |
| --- | --- | --- | --- | --- | --- |
|  |  | **Model 1** | | **Model 2** | |
|  | **Predictors** | **Estimates (95% Confidence Interval)** | **p-value** | **Estimates (95% Confidence Interval)** | **p-value** |
| **SKIPOGH 1** (N=441) | Intercept | 6.89 (6.51; 7.26) | **<10^-3^** | 7.44 (6.80; 8.08) | **<10^-3^** |
|  | Espresso ^b^ | 0.10 (0.05; 0.15) | **<10^-3^** | -0.01 (-0.11; 0.09) | 0.82 |
|  | Change of slope |  |  | -0.90 (-1.48; -0.32) | **0.002** |
|  | Change of slope ˣ espresso ^b^ |  |  | 0.33 (0.19; 0.48) | **<10^-3^** |
| **SKIPOGH 2** (N=619) | Intercept | 6.49 (6.17; 6.81) | **<10^-3^** | 7.25 (6.36; 8.15) | **<10^-3^** |
|  | Espresso ^b^ | 0.09 (0.05; 0.13) | **0.007** | -0.03 (-0.16; 0.10) | 0.66 |
|  | Change of slope |  |  | -0.81 (-1.68; 0.06) | 0.07 |
|  | Change of slope ˣ espresso ^b^ |  |  | 0.14 (-0.01; 0.28) | 0.07 |

^a^ Log transformed

^b^ Reported number of espresso cups consumed per day.

ˣ Interaction term

Model 1 was not adjusted for the change of slope, while Model 2 was adjusted for change of slope at 3 and 4 cups of espresso per day in SKIPOGH 1 and 2, respectively. All models were adjusted for age, smoking and time spent between last caffeine intake and blood drawing.

SKIPOGH 1 and 2 had 10% and 9% increase in log plasma levels after each espresso cup, ignoring the change of the slope (Model 1). Plasma SKIPOGH1 and 2 levels were 1086 (exp(6.89+0.10)) and 721 (exp(6.49+0.09)) ng/ml for a consumption of one espresso per day.

Considering the change of the slope (Model 2), log plasma levels increased by 33% for 3 espresso cups in SKIPOGH1 and 14% for 4 in SKIPOGH2. Thus, SKIPOGH1 plasma levels were 2369 (exp(7.44+0.33)) after 3 espresso cups, whereas SKIPOGH2 plasma levels were 1620 (exp(7.25+0.14)) after 4 cups. In both waves, more espresso consumption did not raise log plasma caffeine and its metabolites.

**Supplementary Figure 2**: Correlation between log paraxanthine/caffeine ratios (reflecting CYP1A2 activity) and self-reported caffeine daily consumption in SKIPOGH


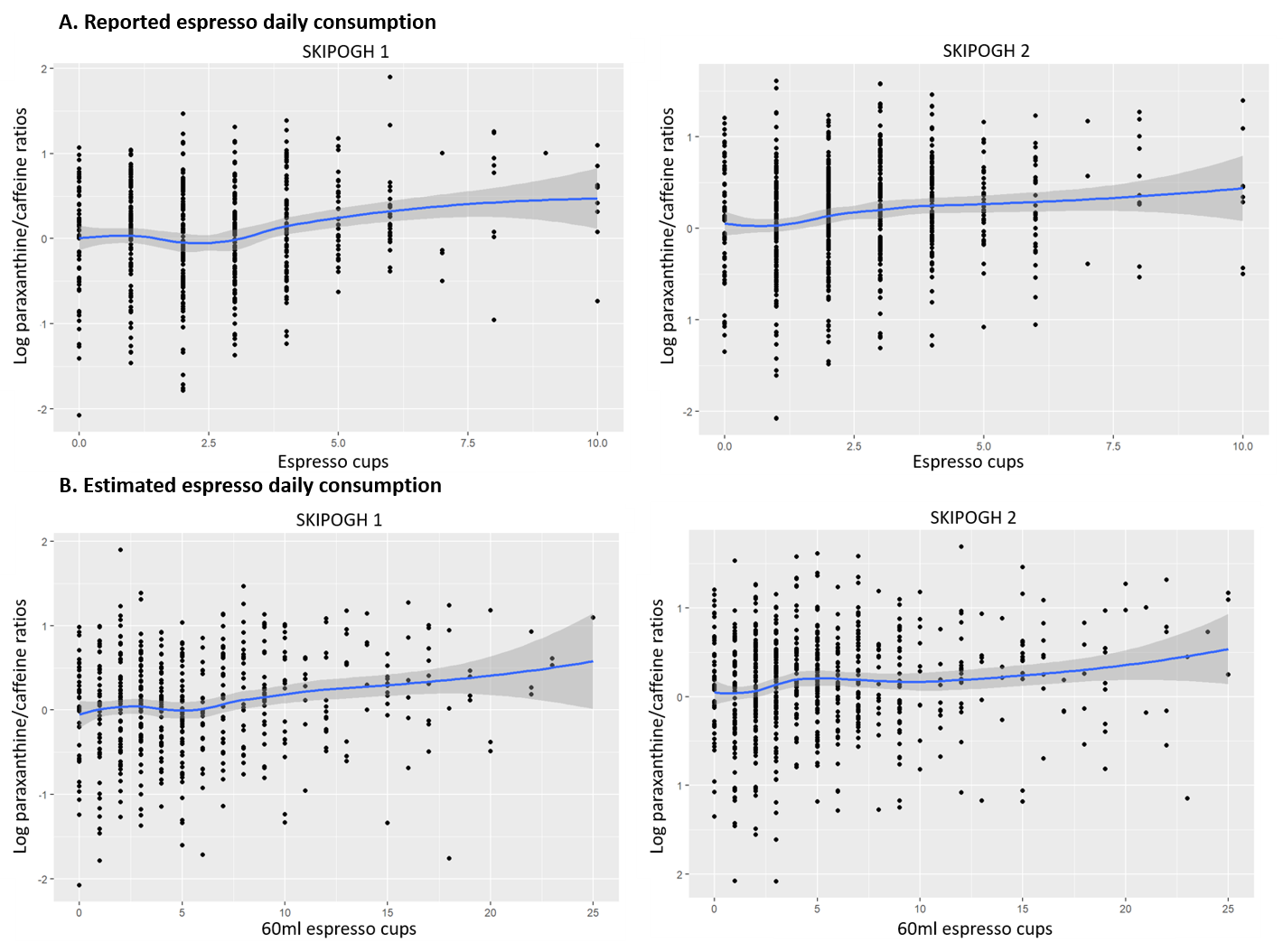


**Supplementary Table 4:** Association between paraxanthine/caffeine ratios (reflecting CYP1A2 activity) and self-reported caffeine consumption in SKIPOGH (1 and 2) and CoLaus|PsyCoLaus studies

|  |  | **Paraxanthine/caffeine ratio ^g^** | |
| --- | --- | --- | --- |
|  | **Predictors** | **Estimates (95% confidence Interval)** | **p-value** |
| **SKIPOGH 1^a^** | **Espresso** (N=441)^c^ | 0.05 (0.02; 0.08) | **<10^-3^** |
|  | Intercept | -0.01 (-0.24; 0.21) | 0.91 |
|  | **60ml espresso** (N=428)^d^ | 0.01 (0.00; 0.03) | **0.01** |
|  | Intercept | -0.00 (-0.25; 0.24) | 0.97 |
| **SKIPOGH 2^a^** | **Espresso** (N=619)^c^ | 0.05 (0.02; 0.07) | **<10^-3^** |
|  | Intercept | 0.12 (-0.06; 0.31) | 0.20 |
|  | **60ml espresso** (N=610)^d^ | 0.01 (0.00; 0.02) | **0.02** |
|  | Intercept | 0.16 (-0.03; 0.36) | 0.10 |
| **CoLaus\| PsyCoLaus^b^** | **Number of coffee cups** (N=4455)^e^  Intercept(2 or more per day)  None in the last 4 weeks  1-3 per month  1-2 per week  3-4 per week  1/day | 0.87 (0.75; 1.00)  -0.14 (-0.20; -0.08)  -0.15 (-0.24; -0.05)  -0.19 (-0.29; -0.09)  -0.17 (-0.24; -0.09)  -0.14 (-0.24; -0.10) | **<10^-3^**  **<10^-3^**  **0.003**  **<10^-3^**  **<10^-3^**  **<10^-3^** |
|  | **Number of caffeinated beverage cups** (N=4423)^f^  Intercept (more than 6 per day)  None  1-3  4-6 | 0.60 (0.47; 0.72)  -0.22 (-0.32; -0.11)  -0.15 (-0.24; -0.07)  -0.05 (-0.14; 0.04) | **<10^-3^**  **<10^-3^**  **0.001**  0.26 |

^a^ Models were adjusted for age, smoking and time spent between last caffeine intake and blood intake.

^b^ Models were adjusted for age, sex and smoking.

^c^ Considering only the reported number of espresso consumption.

^d^ Considering all caffeine source listed in the SKIPOGH questionnaire translated into number of 60 ml espresso.

^e^ Compared to those who reported consuming at least 2 cups of coffee per day.

^f^ Compared to those who reported consuming more than 6 cups of caffeinated beverages per day.

^g^ Log transformed

Example for result interpretation: Considering a participant in SKIPOGH1, for a reported consumption of one espresso, paraxanthine/caffeine ratios were 1.04 (exp(-0.01+0.05)) and, for a reported consumption of one espresso per day by a SKIPOGH 2 participant, paraxanthine/caffeine ratios were 1.19 (exp(0.12+0.05)).

**Supplementary Table 5:** Association between log plasma levels of caffeine and its metabolites and the self-reported number of cups of coffee consumed in CoLaus|PsyCoLaus

|  | | Caffeine + paraxanthine + theophylline plasma levels ^a^ | |
| --- | --- | --- | --- |
|  | **Predictors** | **Estimates (95% Confidence Interval)** | **p-value** |
| **coffee consumption** (N=4455) | Intercept (2 or more coffee cups per day) | 6.79 (6.61; 6.97) | **<10^-3^** |
|  | Cups of coffee consumed ^b^  None in the last 4 weeks  1-3 per month  1-2 per week  3-4 per week  1/day | -1.21 (-1.32; -1.10)  -1.24 (-1.43; -1.06)  -0.72 (-0.91; -0.53)  -0.48 (-0.62; -0.34)  -0.26 (-0.34; -0.18) | **<10^-3^**  **<10^-3^**  **<10^-3^**  **<10^-3^**  **<10^-3^** |

^a^ Log transformed .

^b^ Compared to those who reported consuming at least 2 cups per day.

Model was adjusted for age and smoking.

**Supplementary Figure 3**: Associations between log paraxanthine/caffeine ratios (reflecting CYP1A2 activity) and self-reported caffeine consumption in CoLaus|PsyCoLaus


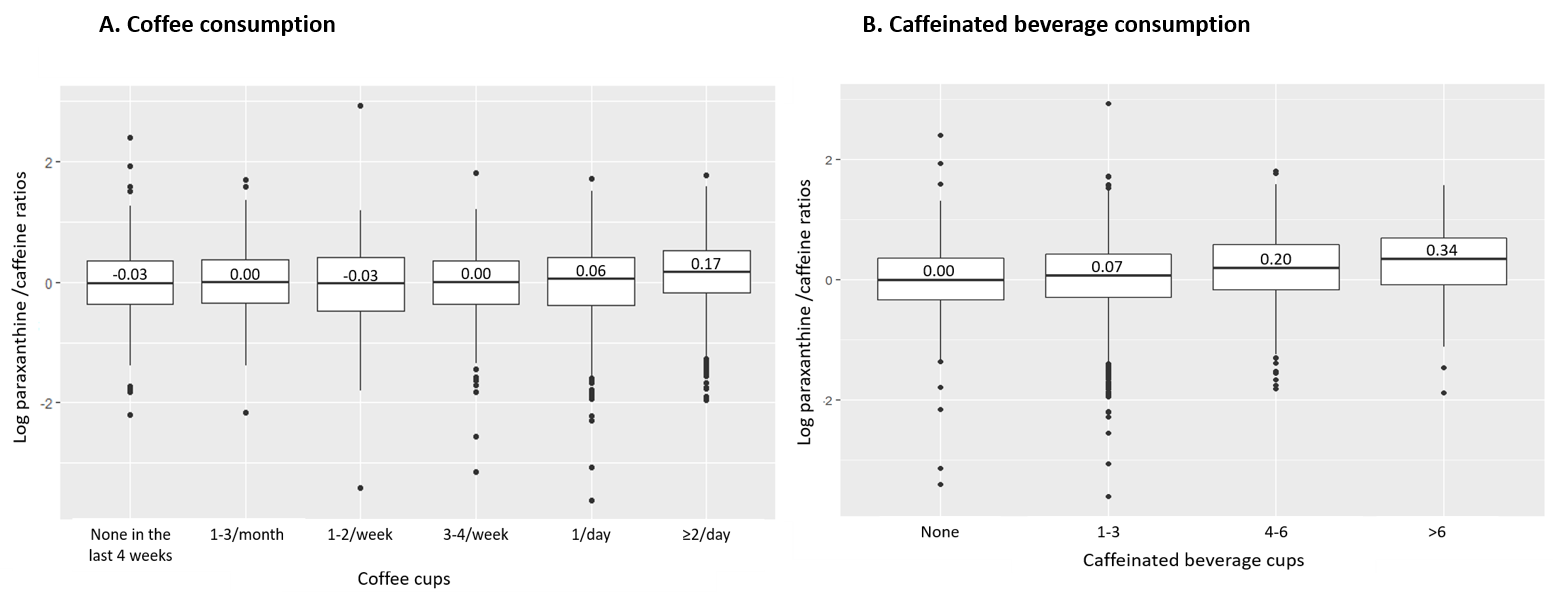


**Supplementary Figure 4**: Distribution of log plasma levels of caffeine and its two metabolites according to the self-reported number of coffee cups consumed in CoLaus|PsyCoLaus (N=4455)


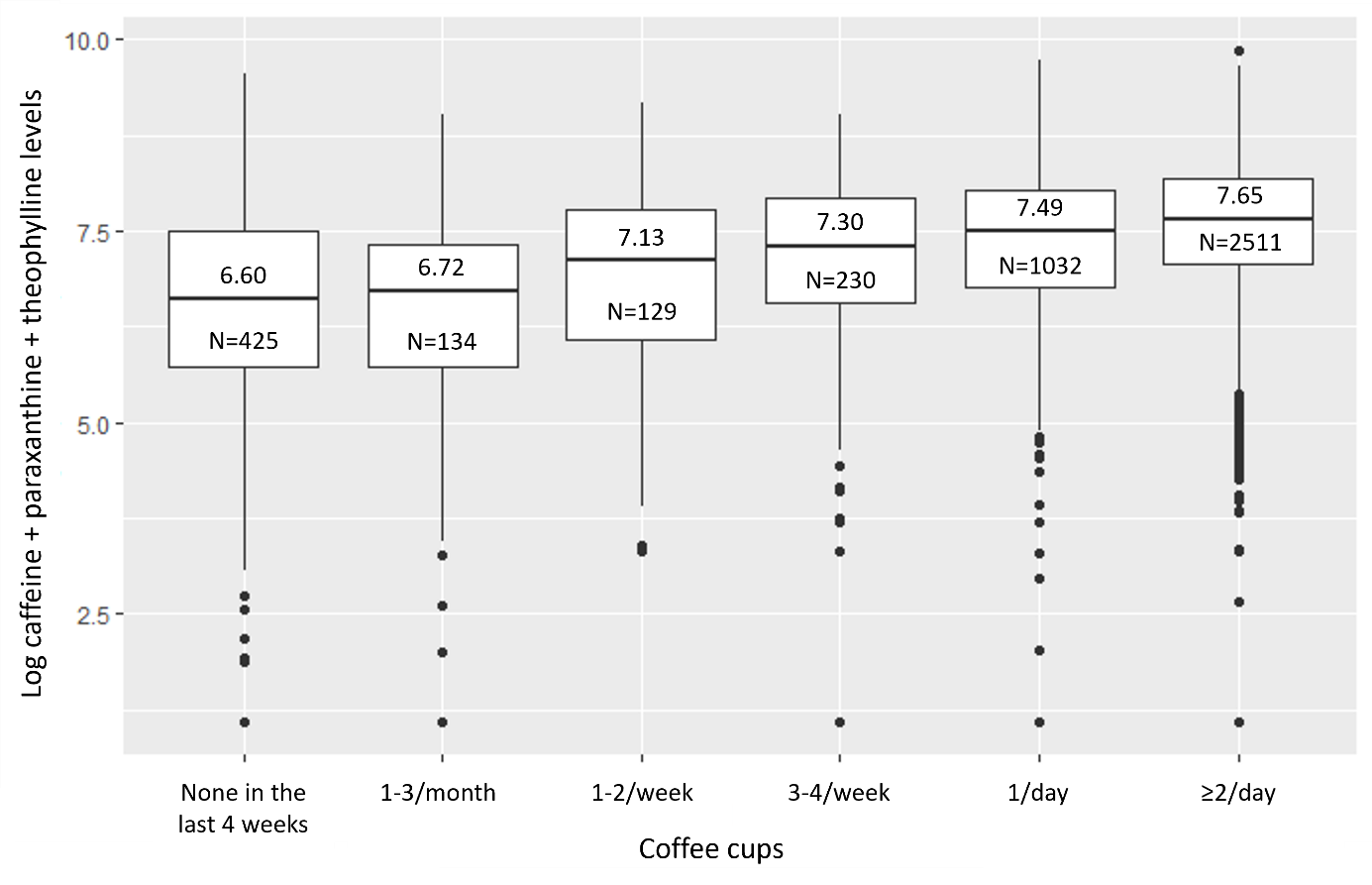


**Supplementary Table 6:** Pairwise comparison of log plasma levels of caffeine and its two metabolites according to the coffee consumption frequency in CoLaus|PsyCoLaus (p-value)

|  | **None in the last 4 weeks** | **1-3/month** | **1-2/week** | **3-4/week** | **1/day** |
| --- | --- | --- | --- | --- | --- |
| **1-3/month** | 0.25 |  | | | |
| **1-2/week** | 0.003 | 0.003 |  | | |
| **3-4/week** | <10^-4^ | <10^-4^ | 0.04 |  | |
| **1/day** | <10^-4^ | <10^-4^ | <10^-3^ | 0.01 |  |
| **≥2/day** | <10^-4^ | <10^-4^ | <10^-4^ | <10^-4^ | <10^-4^ |

Comparison were conducted using Dunn’s test
